# Chinese expert consensus on diagnosis and treatment of trauma-induced hypercoagulopathy

**DOI:** 10.1186/s40779-021-00317-4

**Published:** 2021-04-12

**Authors:** Jing-Chun Song, Li-Kun Yang, Wei Zhao, Feng Zhu, Gang Wang, Yao-Peng Chen, Wei-Qin Li, Chinese People’s Liberation Army Professional Committee of Critical Care Medicine and Chinese Society of Thrombosis, Hemostasis and Critical Care, Chinese Medicine Education Association

**Affiliations:** 1 Department of Critical Care Medicine, the 908th Hospital of Joint Logistics Support Forces of Chinese PLA, Nanchang, 330002 China; 2 Department of Neurosurgery, the 904th Hospital of Joint Logistics Support Forces of Chinese PLA, Wuxi, 214044 Jiangsu China; 3 Division of Vascular and Interventional Radiology, Nanfang Hospital, Southern Medical University, Guangzhou, 510515 China; 4 Department of Critical Care Medicine, Shanghai East Hospital, Tongji University, Shanghai, 200120 China; 5 Department of Critical Care Medicine, the Second Affiliated Hospital of Xi’an Jiaotong University, Xi’an, 710001 China; 6 Department of Blood Transfusion, the 923th Hospital of Joint Logistics Support Forces of Chinese PLA, Nanning, 530021 China; 7 Department of Critical Care Medicine, General Hospital of Eastern Theater Command of Chinese PLA, Nanjing, 210002 China

**Keywords:** Trauma, Coagulation dysfunction, Thrombosis, Diagnosis, Treatment

## Abstract

Trauma-induced coagulopathy (TIC) is caused by post-traumatic tissue injury and manifests as hypercoagulability that leads to thromboembolism or hypocoagulability that leads to uncontrollable massive hemorrhage. Previous studies on TIC have mainly focused on hemorrhagic coagulopathy caused by the hypocoagulable phenotype of TIC, while recent studies have found that trauma-induced hypercoagulopathy can occur in as many as 22.2–85.1% of trauma patients, in whom it can increase the risk of thrombotic events and mortality by 2- to 4-fold. Therefore, the Chinese People’s Liberation Army Professional Committee of Critical Care Medicine and the Chinese Society of Thrombosis, Hemostasis and Critical Care, Chinese Medicine Education Association jointly formulated this Chinese Expert Consensus comprising 15 recommendations for the definition, pathophysiological mechanism, assessment, prevention, and treatment of trauma-induced hypercoagulopathy.

## Background

Trauma causes at least 5.8 million deaths every year in the whole world, which account for 9% of annual deaths [1]. Trauma-induced coagulopathy (TIC) is an independent risk factor for poor prognosis of trauma patients [2, 3]. According to the recommendation of the International Society on Thrombosis and Hemostasis, TIC is defined as coagulation dysfunction caused by post-traumatic tissue injury, and manifests as hypercoagulability that leads to thromboembolism or hypocoagulability that leads to uncontrollable massive hemorrhage [4]. Previous studies have mainly focused on hemorrhagic coagulopathy and uncontrolled bleeding caused by the hypocoagulable phenotype of TIC [5], while it has recently been shown that the incidence of trauma-induced hypercoagulopathy can reach 22.2–85.1% in trauma patients, increasing thrombotic events and mortality by 2–4 times [3, 6, 7]. Therefore, the People’s Liberation Army Professional Committee of Critical Care Medicine and the Chinese Society of Thrombosis, Hemostasis and Critical Care formed a committee of experts, who met initially in November 2020 and eventually reached a consensus concerning the definition, pathophysiological mechanism, assessment, prevention, and treatment of trauma-induced hypercoagulopathy. The consensus includes 15 recommendations, which may help guide relevant clinical research and practice.

### Definition

#### Recommendation 1: trauma-induced hypercoagulopathy is a coagulation disorder caused by post-traumatic tissue injury, and it manifests as pathological hypercoagulability causing a prothrombotic condition or thromboembolism

Rapid blood coagulation was first described in 1772, while the association of blood loss with hypercoagulability was first reported in 1955. In 1964, Innes et al. [8] monitored serial changes in coagulation and fibrinolysis among 42 trauma patients after admission, and found that the clotting time was significantly shortened in the early stage of trauma. During trauma, blood enters a moderate hypercoagulable state to achieve rapid hemostasis. However, excessive blood clotting and post-traumatic imbalance of coagulation system may promote trauma-induced hypercoagulopathy [4, 9], a TIC hypercoagulable phenotype characterized by endothelial injury, excessive thrombin generation, hyperfibrinogenemia, platelet hyperactivity, anticoagulant pathways impairment and fibrinolysis shutdown [10].

Thrombotic complications such as venous thrombosis, arterial thrombosis, and microthrombosis may occur in patients with trauma-induced hypercoagulopathy, but the location of thrombus deposition depends on the blood flow turbulence, hypercoagulable state, vascular injury site, and original pathological changes such as atherosclerosis and angionoma [11]. Venous thrombosis is more common in the femoral, internal iliac, and intermuscular veins, than in the subclavian, intracranial, portal, and splenic veins [12, 13]. Particularly, venous thromboembolism (VTE) has been defined as a disease that encompasses two primary entities, deep venous thrombosis (DVT) and pulmonary embolism. In contrast, arterial thrombosis often occurs in pulmonary, intracranial, and coronary arteries, while it can sometimes be found in the aorta as well as peripheral, carotid, splenic, and mesenteric arteries [14].

#### Recommendation 2: the incidence of trauma-induced hypercoagulopathy depends on the patient’s sex, age, and weight, as well as the type and severity of injury

Thromboelastography (TEG) of 464 patients (23% female) with injury severity scores (ISS) > 15 showed that α angle, an index of the dynamics of clot formation, was an average of 3° larger in women than men, while maximum amplitude (MA), a measure of clot strength, was an average of 3 mm larger in women [15]. This may help explain why after trauma, women show a significantly more hypercoagulable state and lower mortality rate than men. Another study on 7194 trauma patients showed that the VTE incidence in patients younger than 13 years remained below 1.5%, but gradually increased with age: it was 2.3% for patients older than 13 years, 5.1% for patients older than 15 years, 5.5% for patients 31–35 years, and 7.6% for patients 46–50 years. However, VTE risk was lower among patients older than 50 years than among younger patients [16]. In a prospective study of 687 trauma patients to examine the relationship between obesity [body mass index (BMI) ≥ 30 kg/m^2^] and post-injury hypercoagulability, obese patients showed higher MA values, lower fibrinolysis [clot lysis achieved 30 min after MA (LY30%)], and higher incidence of VTE than non-obese patients [17]. A study of 72 morbidly obese patients undergoing bariatric surgery showed that the average BMI of obese patients decreased from 44.6 kg/m^2^ to 31.4 kg/m^2^ by 6 months after surgery. After surgery, patients also showed longer time to clot initiation (TEG R-time; 1.3 s on average), lower MA (2.4 mm on average), and significantly greater sensitivity to tissue-type plasminogen activator (t-PA) than before surgery [18].

Serial TEG on 95 blunt trauma patients without surgery indicated that all patients were hypercoagulable, 58% were in a state of fibrinolysis shutdown, and 50% showed resistance to t-PA [19]. Another study showed that VTE incidence among 603 patients with severe traumatic brain injury was 19.7%, and it usually occurred within 6 days after admission [20]. Serial TEG of 118 trauma patients also revealed an association between injury severity and trauma-induced hypercoagulability [6]. The number of hypercoagulable patients in that study gradually increased with increasing ISS, and the highest hypercoagulopathy incidence (38.7%) was observed among patients with ISS 6–15. However, the number of hypercoagulable patients gradually decreased with further increase of the ISS. In addition, a competing risks analysis of 2370 trauma patients showed that 11.2% developed DVT and that 38% of DVT cases occurred within 48 h after admission, implying that severe injury (ISS ≥ 15) is an independent risk factor for DVT [21].

## Pathophysiological mechanism

### Recommendation 3: tissue damage, inflammation and stress are the main pathophysiological mechanisms of trauma-induced hypercoagulopathy

**Fig. 1:**
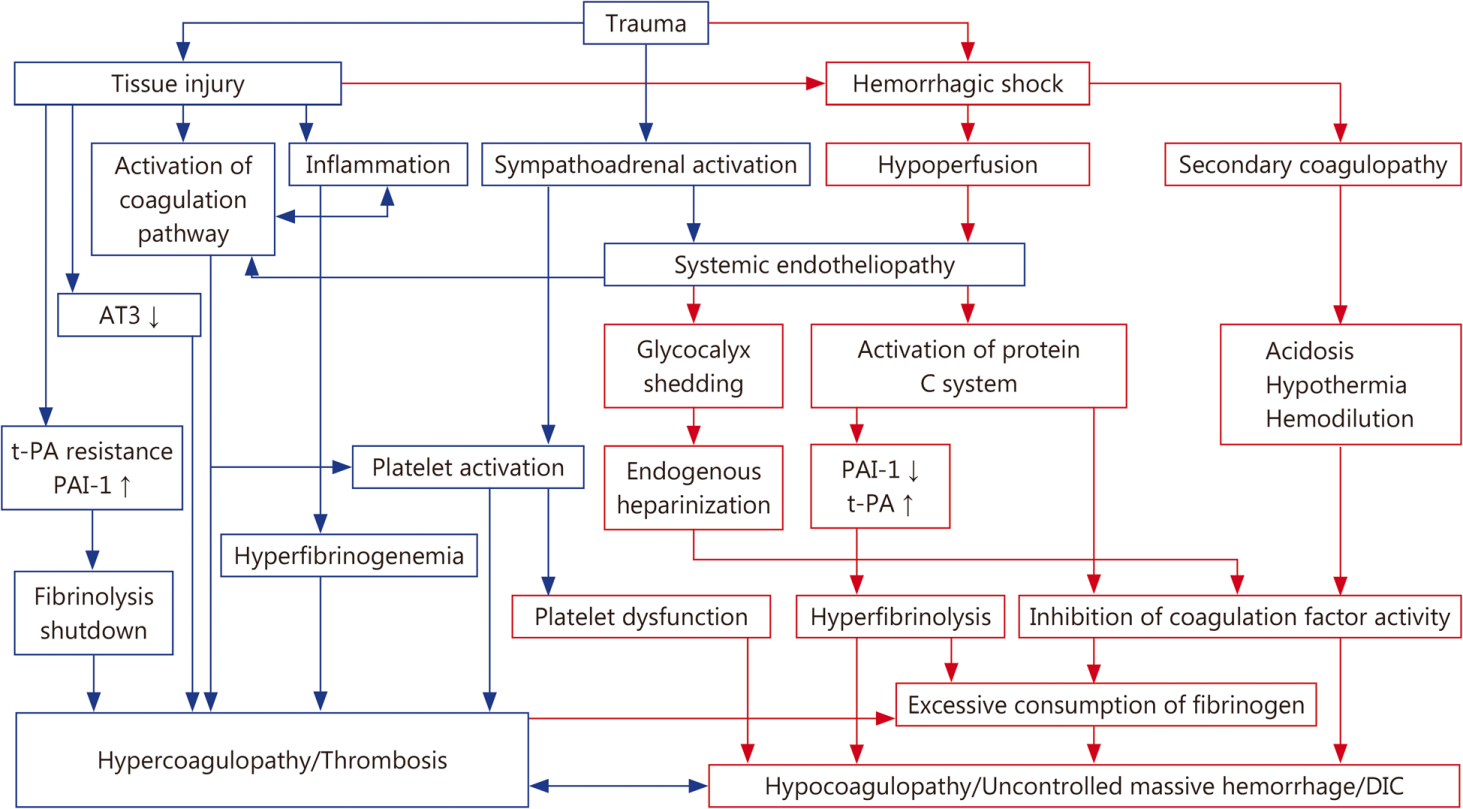
Pathophysiological mechanism of TIC. The blue box represents hypercoagulable mechanism, and the red box represents hypocoagulable mechanism. TIC, trauma-induced coagulopathy

During tissue injury, the transmembrane glycoprotein tissue factor is released in large amounts, activating extrinsic coagulation factors and platelets, in turn triggering and expanding massive thrombin generation and promoting thrombosis [22–24] (Fig. 1). High circulating actin and myosin released due to cell death can also promote clot propagation and fibrinolysis shutdown, reflected in low TEG LY30%, high α angle, and elevated levels of plasminogen activator inhibitor type-1 (PAI-1) [25, 26]. Moreover, uncontrolled systemic release of endogenous damage-associated molecular patterns (DAMPs) after trauma triggers inflammasomes to activate caspases, which leads in turn to the release of interleukin-1β and interleukin-18 as well as promotes pyroptosis [27]. Simultaneously, numerous inflammatory mediators activate the coagulation system and amplify coagulation disorders through the crosstalk between inflammation and coagulation [28]. At the same time, numerous inflammatory mediators activate the coagulation system and enhance coagulation disorders through crosstalk between inflammation and coagulation [28]. Trauma-induced inflammation may also stimulate a continuous increase in plasma fibrinogen levels and promote heparin resistance, contributing to clot formation [29]. Further studies have shown that sympathetic hyperactivity and catecholamine release induced by traumatic stress promote endotheliopathy [30]. TF released after endothelial damage can activate the coagulation pathway, increase the activity of factor VII, and enhance thrombin-induced platelet aggregation, leading to hypercoagulability [31].

### Recommendation 4: TIC is characterized by a phenotypic transformation between hypercoagulability and hypocoagulability

An early study showed that prothrombin time (PT) and activated partial prothrombin time (APTT) are shortened in hemorrhagic shock patients but prolonged in refractory shock patients [32]. A study of 42 injury patients based on serial coagulation and fibrinolysis assays [8] showed that clot lysis and clotting time accelerated in the first hours after trauma, after which fibrinolysis and clotting time prolonged. Although the platelet count and plasma fibrinogen levels decreased for about 3 days, they subsequently increased and remained at unusually high levels for 1 week. Further studies have confirmed that increased levels of endogenous thrombopoietin promote thrombocytosis after trauma [33, 34].

**Fig. 2:**
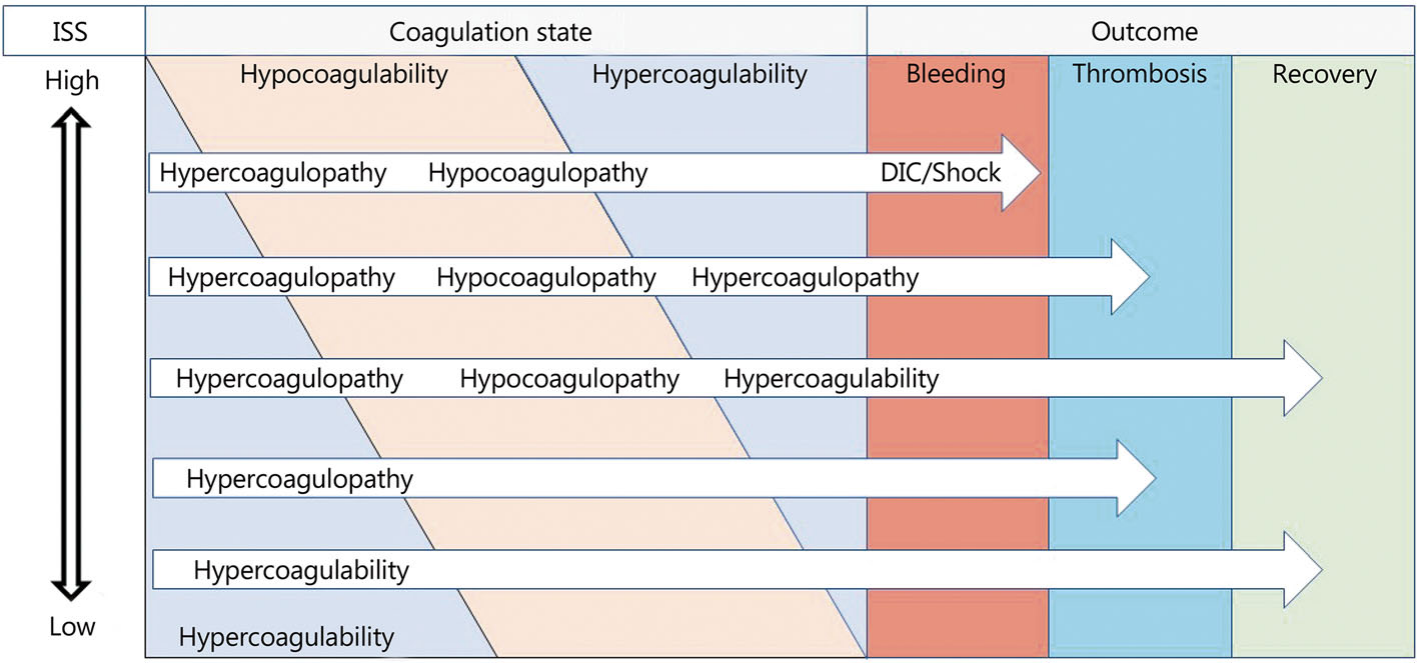
TIC phenotypes transformation between hypercoagulability and hypocoagulability

Trauma patients with low ISS may develop hypercoagulability to facilitate hemostasis and rehabilitation. However, if venous, arterial, or microvascular thrombosis develops at the same time, the patient is considered to have trauma-induced hypercoagulopathy [3, 4]. With increasing ISS and blood loss, the proportion of hypercoagulability in trauma patients gradually decreased and the proportion of hypocoagulability increased accordingly. Hypercoagulable phenotype may transform into hypocoagulable phenotype in the patients with continuous bleeding. These patients may return to a hypercoagulable or normocoagulable state within 48 h if bleeding stops [35]. However, in the event of uncontrollable bleeding, the hypocoagulable phenotype will develop into disseminated intravascular coagulation (DIC) (Fig. 2).

The pathophysiological mechanism of the hypercoagulable-to-hypocoagulable phenotype transformation is shown in Fig. 1. Tissue hypoperfusion induced by traumatic hemorrhagic shock can cause endothelial injury and glycocalyx shedding as well as activate the protein C pathway, while secondary endogenous heparinization can inhibit the activity of coagulation factors [36, 37]. Injury and swelling of vascular endothelial cells lead to the enlargement of the endothelial cell gap and the development of capillary leakage syndrome, thus exacerbating tissue hypoperfusion and coagulation disorders [30, 38]. Moreover, activated protein C released by endothelial cells can inhibit PAI-1, leading to hyperfibrinolysis; and it can inhibit factors V and VIII, leading to hypocoagulability [39]. The massive blood loss and fluid resuscitation can then induce hemodilution, acidosis, and hypothermia, which in turn lead to secondary coagulopathy [40]. The inflammatory responses induced by tissue injury and hypoperfusion lead to platelet exhaustion after the adenosine diphosphate (ADP)-induced release of massive granules [41], while hyperfibrinolysis and the consumption of coagulation factors promote hypofibrinogenemia, thus contributing to coagulation dysfunction and increasing risk of mortality in trauma patients [42].

### Assessment

#### Recommendation 5: routine coagulation tests are recommended for screening patients for trauma-induced hypercoagulopathy

Routine coagulation tests include: 1) assessment of the exogenous coagulation system based on measurement of PT or the International Standardized Ratio (INR); 2) assessment of the endogenous coagulation system based on measurement of APTT; 3) assessment of the common coagulation pathways based on measurement of thrombin time (TT) and fibrinogen levels; 4) assessment of the fibrinolytic system based on assays of D-dimers (DD) and fibrinogen (or fibrin) degradation products (FDPs); and 5) estimation of the platelet count. Previous studies have recommended that patients whose PT > 18 s, INR > 1.5, APTT >60s, or TT > 15 s can be diagnosed as hypocoagulable phenotype of TIC without the influence of anticoagulant drugs or abnormal blood specimens [43]. In contrast, no diagnostic criteria have yet been reported for the hypercoagulable phenotype of TIC based on routine coagulation tests; this reflects the insensitivity of the conventional coagulation indexes to hypercoagulability [44, 45]. However, these tests can still indicate trauma-induced hypercoagulopathy. In particular, shortened APTT and PT are indicators of the coagulation factor hyperfunction, suggesting the occurrence of hypercoagulability [46]. In addition, hyperfibrinogenemia contributes to post-traumatic hypercoagulability, which is defined by a plasma fibrinogen concentration > 4.0 g/L [29, 47], while thrombocytosis contributes to post-injury hypercoagulability defined by a platelet count >400 × 10^9^/L [48, 49]. Nevertheless, DD and FDPs levels are not efficient indicators of trauma-induced hypercoagulopathy, as they are commonly elevated during secondary fibrinolysis after post-injury thrombosis [50]. In contrast, the thrombin–antithrombin complex is a sensitive marker of thrombin formation and, combined with other indicators, it can reliably predict blood hypercoagulability [51].

#### Recommendation 6: viscoelasticity coagulation tests are recommended for the diagnosis of trauma-induced hypercoagulopathy

Point of care assays of viscoelastic coagulation can comprehensively and accurately evaluate the coagulation state by taking whole blood as test sample [52–54]. Therefore, it is recommended that viscoelastic tests are used to evaluate the coagulation function of severe trauma patients and guide replacement therapy [5, 55]. Viscoelastic coagulation tests involve TEG^@^, rotation thromboelastometry (ROTEM^@^), and coagulation and platelet function analysis (Sonoclot^@^ or Centuryclot^@^). The main indices of TEG^@^ include R-time for coagulation factor activity, α angle and K-time for fibrinogen function, MA for platelet function and LY30% for fibrinolytic function. The hypercoagulability may be implied by shortened R-time for coagulation factors hyperfunction, increased α - angle and shortened K-time for fibrinogen hyperfunction, increased MA for platelet hyperfunction and LY30% < 0.8% for fibrinolysis shutdown. The main indicators of Sonoclot^@^ or Centuryclot^@^ include activated clotting time (ACT) for coagulation factor function, clot rate (CR) for fibrinogen function and PF for platelet function. The hypercoagulability may be identified by shortened ACT for coagulation factors hyperfunction, increased CR for fibrinogen hyperfunction and increased PF for platelet hyperfunction [56].

Although 35 TEG-based criteria have been reported for the diagnosis of hypercoagulopathy, the diagnostic criteria for trauma-induced hypercoagulopathy have yet not been certified. A meta-analysis of 1893 studies on the relationship between TEG and post-injury hypercoagulability and thrombosis suggested that MA > 66.7 mm could be used as a diagnostic criterion for trauma-induced hypercoagulopathy [57]. In addition, a retrospective study of 983 trauma patients found that 582 (85.1%) patients developed hypercoagulοpathy at admission, while 99 (14.5%) of them were diagnosed with DVT by ultrasound scan. The incidence of DVT was significantly higher in trauma patients with hypercoagulopathy based on TEG than in patients without hypercoagulopathy [odds ratio (*OR*) 2.41, 95% confidence interval (CI) 1.11–5.24, *P* = 0.026] [7].

#### Recommendation 7: Doppler ultrasound is recommended as a routine method for detecting thrombotic complications associated with trauma-induced hypercoagulopathy

The thrombotic risk assessment scale need not be used with patients who have trauma-induced hypercoagulopathy, because the scale always classifies such patients as being at high risk [58]. Instead, thrombus examination can be performed using radiology or Doppler ultrasonography, a non-invasive and reproducible method that does not require contrast agents or patient handling [59]. Doppler ultrasonography can accurately diagnose thrombosis in deep blood vessels of the lower extremities, as well as in neck, abdominal organ, and cardiopulmonary blood vessels, making it suitable for detecting thrombotic complications in patients with trauma-induced hypercoagulopathy. If necessary, computed radiography, magnetic resonance, and digital subtraction angiography can be further used to accurately diagnose thrombosis in brain, lung, and other organs [59]. However, risks of these imaging modalities due to patient transport, injection of contrast agent, and invasive procedures should be considered in light of the patient’s condition.

#### Recommendation 8: thrombophilia testing is recommended for patients with recurrent post-traumatic thromboembolism

Thrombophilia is a pathological state with high thromboembolic tendency due to genetic deficiency, acquired defects or risk factors. Hereditary thrombophilia is induced by 1) deficiencies of antithrombin, protein C, or protein S; 2) deficiencies of coagulation factors manifesting as prothrombin G20210A mutation, abnormal fibrinogenemia, or activated protein C resistance; 3) fibrinolysin deficiency manifesting as hypoplasminogenemia, t-PA deficiency, and increased PAI-1 levels; 4) hyperhomocysteinemia, a defect associated with homocysteine metabolism; and 5) increased levels of coagulation factors [60]. In contrast, acquired thrombophilia can develop due to myeloproliferative tumor, antiphospholipid syndrome, paroxysmal nocturnal hemoglobinuria, nephrotic syndrome, or inflammatory bowel disease. Acquired risk factors of thrombophilia include pregnancy,surgery, long-term bed rest, oral contraceptives, hormone replacement therapy, and trauma-induced hypercoagulopathy. Trauma patients with thrombosis need not be routinely examined for hereditary thrombophilia [61]. In contrast, thrombotic events are 2–20 times more likely to occur in trauma patients with other types of thrombophilia [62], so thrombophilia testing is recommended for patients with recurrent post-traumatic thromboembolism or family history of thrombotic disease [63] (Table 1).

**Table 1:** Thrombophilia testing

Classification	Disease	Workup
Hereditary thrombophilia	AT deficiency	AT activity, antigen
	Protein C deficiency	Protein activity, antigen
	Protein S deficiency	Protein S activity, Free protein S antigen
	Prothrombin G20210A	Genetic testing
	APC resistance	Activated protein C resistance test, Genetic testing
Acquired thrombophilia	APLS	clinical, vascular thrombosis and/or pregnancy morbidity; laboratory, 1 of the following on ≥2 occasions at least 12 weeks apart: IgG or IgM anti-cardiolipin antibodies (>40 U); IgG or IgM anti-β2-glycoprotein I antibodies (> 40 U); LA
	PNH	CBC, haptoglobin, LDH, total/direct bilirubin, iron studies, urinalysis, peripheral blood flow cytometry
	Malignant tumor, SLE, nephrotic syndrome, collagen angiopathy, inflammatory bowel disease, obesity	History, physical examination, blood and urine routine examination, hepatic and renal functions, chest radiograph, tumor screening
	Myeloproliferative neoplasm	Mutation analysis for JAK2, calreticulin and thrombopoietin receptor MPL

*AT* antithrombin, *APC* activated protein C, *APLS* antiphospholipid antibody syndrome, *CBC* complete blood count, *LA* lupus anticoagulant, *LDH* lactate dehydrogenase, *PNH* paroxysmal nocturnal hemoglobinuria, *SLE* systemic lupus erythematosus, *JAK* janus kinase

### Prevention

#### Recommendation 9: iatrogenic factors causing thrombosis should be avoided in patients with trauma-induced hypercoagulopathy

Hemostatic drugs can reduce bleeding in trauma patients, but excessive dosage or extended drug administration may lead to acquired thrombosis. Tranexamic acid (*trans*-4-aminomethylcyclohexane-1-carboxylic acid; TXA) is a synthetic lysine analogue that can competitively bind to the lysine binding site of plasminogen and prevent its interaction with fibrin. In the CRASH-2 (clinical randomization of an antifibrinolytic in significant hemorrhage) trial, 20,211 trauma patients were randomly treated with TXA or placebo [64]. Specifically, patients in the TXA group (*n* = 10,096) received a loading dose of 1 g TXA intravenously over 10 min, followed by 1 g TXA over 8 h. Risk of all-cause death was significantly lower in the TXA group than in the placebo group (14.5% vs. 16.0%), as was death due to bleeding (4.9% vs. 5.7%), but there was no difference in the incidence of thrombotic events (1.7% vs. 2.0%). The retrospective MATTERs (military application of tranexamic acid in trauma emergency resuscitation) study demonstrated that the rate of all-cause mortality among patients who underwent emergency resuscitation in military combat was significantly lower among those who received TXA than those who did not (17.4% vs. 23.9%), but VTE incidence in the TXA group was about 10 times that in the untreated group [65]. A retrospective cohort study of 549 combat casualties admitted to US military hospitals from October 2010 to November 2012 also indicated that TXA was an independent risk factor for the development of VTE [66]. According to a retrospective analysis of 3773 military casualties, the use of TXA was not associated with mortality but did increase the risk of VTE [67]. TXA overuse can be defined as the administration of TXA to a hemodynamically stable trauma patient, while TXA underuse can be defined as failing to administer TXA, or delaying its administration, to a trauma patient who receives a massive blood transfusion. Both TXA overuse and underuse were observed in US military combat support hospitals in Afghanistan [68]. Therefore, hemostatic drugs should be used rationally in order to prevent iatrogenic thrombosis in patients with trauma-induced hypercoagulopathy.

Repeated vascular puncture and catheter implantation during trauma treatment are also independent risk factors for thrombosis, especially in pediatric trauma patients [69, 70]. However, both can cause vascular endothelial injury, activate the coagulation system, and promote thrombosis at sites of injury in blood vessels. In severe cases, pseudoaneurysm, arteriovenous fistula, intimal denudation, and even vascular rupture may occur [71]. Therefore, to reduce vascular injury, ultrasound-guided vascular puncture may be performed during invasive surgery in trauma patients [72–74].

#### Recommendation 10: mechanical prevention alone or combined with pharmacologic prophylaxis is recommended for preventing thrombosis in patients with trauma-induced hypercoagulopathy

In order to prevent deep vein thrombosis, patients with trauma -induced hypercoagulopathy should receive early rehabilitation when traumatic condition permits [75]. However, the risk of bleeding should first be assessed based on the patient’s characteristics [76], including 1) Patient factors: age ≥ 85 years old, taking oral antiplatelet drugs or anticoagulants. 2) Basic diseases: uncontrolled active bleeding, previous history of intracranial hemorrhage, peptic ulcer, recent stroke, liver and kidney dysfunction. 3) Treatment: planned surgical operation or within 12 h after operation.

For patients at high bleeding risk, mechanical prevention is recommended; for patients at low bleeding risk, mechanical prevention combined with pharmacologic prophylaxis is recommended [77]. One study on the anticoagulation timing of patients with traumatic hypercoagulability suggested that patients who had blunt solid organ injury but had not undergone surgery could initiate VTE chemoprophylaxis if TEG indicated fibrinolytic shutdown (LY30% < 0.5% at 12 h after trauma) or hypercoagulation (MA > 65.8 mm at 24 h after trauma) [19]. One study on the anticoagulation timing of patients with traumatic hypercoagulability suggested that patients who had blunt solid organ injury but had not undergone surgery could initiate VTE chemoprophylaxis if TEG indicated fibrinolytic shutdown (LY30% < 0.5% at 12 h after trauma) or hypercoagulation (MA > 65.8 mm at 24 h after trauma) [19].

Mechanical prevention, including intermittent pneumatic compression and wearing of graded static compression stockings, should be performed in patients at high risk of thrombosis, until the risk factors are eliminated [78]. Proper pressure adjustment and rigorous monitoring are required to avoid complications. Absolute contraindications to mechanical prevention of DVT include lower extremity trauma, lower extremity tissue transplantation, or major limb surgery. Incidence of DVT among trauma patients is significantly lower among those receiving pharmacologic prophylaxis (15–20%) than those not receiving it (20–50%), but craniocerebral injury and fracture are considered independent risk factors for delayed pharmacologic prophylaxis [21]. Moreover, VTE risk is 2–4 times higher in patients with intracranial hemorrhage than in patients with acute ischemic stroke [79]. In contrast, the risk of pulmonary embolism was significantly lower in patients with intracranial hemorrhage receiving low-molecular-weight heparin (LMWH) or unfractionated heparin (UFH) than in patients without pharmacologic prophylaxis in one study [4.2% vs 3.3%; Risk ratio (*RR*) 0.37; 95% CI 0.17–0.80; *P* = 0.01), but no significant effects were observed on the incidence of DVT, hematoma enlargement, or mortality [80, 81]. Moreover, anticoagulants did not aggravate the hematoma and had no significant effects on mortality or the incidence of DVT in those studies. Therefore, intermittent pneumatic compression along with pharmacologic prophylaxis should be applied to patients with intracranial hemorrhage 1) at 48 h after admission if there is no hematoma enlargement or hypocoagulability, 2) at 24 h after craniotomy, or 3) at 72 h after spinal cord injury [82].

In order to reduce the risk of bleeding, subcutaneous injection of LMWH is recommended for patients with normal renal function. The dose of LMWH can be adjusted by monitoring the anti-Xa activity in a target range of (0.2–0.5) IU/mL. In contrast, subcutaneous injection of UFH is recommended for trauma patients with renal dysfunction [83, 84]. Adjustment of the LMWH dose in trauma patients based on the differences in the TEG R-time did not affect the incidence of VTE in the intervention or control groups of one study [85], probably due to the mild condition of the selected patients. This suggests that TEG is insensitive to LMWH treatment. Moreover, among patients who underwent hip and knee arthroplasty, the incidence of DVT among those treated with fondaparinux sodium, a chemically synthesized inhibitor of the Xa factor that does not cause heparin-induced thrombocytopenia (HIT), was 50% lower than the incidence among those receiving enoxaparin, an LMWH anticoagulant (6.8% vs. 13.7%, *P* < 0.001) [86]. For patients at high risk of postoperative thromboembolism and bleeding, the European consensus recommends the administration of low-dose novel anticoagulants (NOACs) at 24 h after surgery [87].

Antiplatelet drugs can also prevent DVT. Weekly follow-up of 74 patients with severe trauma using ROTEM showed that 81% of the patients developed hypercoagulability at admission for 7 days due to platelet hyperfunction [88]. A retrospective analysis of 110 trauma patients showed that the incidence of DVT was significantly lower among patients who took aspirin prior to trauma than among those who did not [89]. The Pulmonary Embolism Prevention study, which enrolled 13,356 hip fracture patients randomly selected from 148 hospitals in Australia, New Zealand, South Africa, Sweden, and the United Kingdom, as well as 4088 hip replacement patients randomly selected from 22 hospitals in New Zealand, also demonstrated that the incidence of VTE in patients receiving aspirin 160 mg /day (*n* = 6679) was 36% lower than that in patients receiving placebo (*n* = 6677) [90].

### Treatments

Thrombosis complicated by trauma-induced hypercoagulopathy can be addressed by treating the primary disease as well as by applying anticoagulant, antiplatelet, interventional, and thrombolytic therapies. However, the site, time, and severity of thrombus formation need to be considered when planning treatment (Table 2).

**Table 2:** Antithrombotic treatment for different thrombotic complications of trauma -induced hypercoagulopathy

Therapy		Venous system				Arterial system			
		DVT	Pulmonary embolism	Portal vein thrombosis	Intracranial venous thrombosis	Miocardial infarction	Ischemic stroke	Peripheral arterial occlusion	Mesenteric arterial embolism
Anticoagulant therapy	UFH	√	√	√	√	√	–	√	√
	LMWH	√	√	√	√	√	–	√	√
	Fondaparinux	√	√	√	√	√	–	√	√
	Warfarin	√	√	√	√	–	–	–	√
	Dabigatran	√	√	√	–	–	–	–	√
	Rivaroxaban	√	√	√	–	–	–	–	√
Antiplatelet therapy	Aspirin	–	–	–	–	√	√	√	√
	Clopidogrel	–	–	–	–	√	√	√	√
	Ticagrelor	–	–	–	–	√	√	√	√
Thrombolytic therapy	r-tPA	√	√	√	–	√	√	–	–
	Urokinase	√	√	√	–	√	√	–	–
Interventional therapy	Catheter-directed thrombolysis	√	√	√	√	√	√	√	√
	Transcatheter thrombus aspiration	√	√	√	√	√	√	√	√
	Mechanical thrombectomy	√	√	√	√	√	√	√	√
	Angioplasty	Cokett syndrome	–	√	–	√	√	√	√
	Stent implantation	Cokett syndrome	–	√	–	√	√	√	√

Cokett syndrome, or common iliac vein compression syndrome, is the obstruction of venous return caused by right common iliac artery compressing left common iliac vein, which can be manifested as left lower limb swelling, superficial varicose veins and DVT.

*DVT* deep venous thrombosis, *UFH* unfractionated heparin, *LMWH* low-molecular-weight heparin.

#### Recommendation 11: reducing stress and tissue damage are prerequisites for improving trauma-induced hypercoagulopathy

Animal experiments showed that epinephrine can accelerate blood clotting, which was confirmed in a study showing that a small epinephrine dose can shorten clotting time to 50–70% of the normal value, whereas a large dose can prolong clotting time [91]. A study of plasma epinephrine and norepinephrine levels in 34 patients undergoing cardiac surgery indicated that stressed patients (given 5 μg/kg fentanyl) had significantly higher levels of both hormones and higher activities of factor VIII and von Willebrand factor than non-stressed patients (given 50 μg/kg fentanyl) [92]. In addition, stress-induced elevated catecholamine concentrations in plasma can damage endothelial cells, activate platelets, inhibit the activity of antithrombin, aggravate inflammation, and promote hypercoagulability [26, 34]. Therefore, reducing stress is recommended to reduce vascular endothelial damage and oxygen consumption, and improve organ perfusion to alleviate hypercoagulability [93].

#### Recommendation 12: bleeding risk needs to be assessed before initiating anticoagulant therapy in patients with trauma-induced hypercoagulopathy

In order to prevent thrombus formation and propagation, anticoagulant therapy is required in patients with trauma-induced hypercoagulopathy. In the GARFIELD-VTE (Global Anticoagulant Registry in the Field of VTE) study between 2014 and 2017, 90.9% of 10,685 patients with VTE received anticoagulant therapy, while thrombolysis, intervention, or surgical treatment was applied to only 5.1% of patients, indicating that anticoagulants are the main treatment for thrombosis [94]. However, before trauma patients receive anticoagulant therapy, their risk of post-traumatic thrombosis and post-therapeutic bleeding should be assessed. Since active bleeding is a contraindication to anticoagulant therapy, patients with potentially life-threatening thrombosis but withoutactive hemorrhage should be treated with anticoagulants as soon as possible after trauma [95].

UFH, LMWH, fondaparinux, argatroban, and bivalirudin are well-known parenteral anticoagulants that act at different steps within the coagulation cascade. UFH is recommended as a first-line treatment due to its short half-life, easy monitoring, and neutralization by protamine. In the treatment of pulmonary embolism, the recommended initial dose of UFH for intravenous administration is 80 U/kg, followed by a maintenance dose of 18 U/ (kg·h) adjusted every 4–6 h based according to the APTT [76]. In the treatment of coronary artery embolism, a loading dose of 60 μg/kg UFH is injected intravenously (≤ 4000 U) along with antiplatelet and thrombolytic therapy, followed by a maintenance dose of 12 U/(kg·h) (≤ 1000 U/h) that is adjusted until the APTT reaches 1.5–2.5 times the control value [96]. After administration of UFH, HIT can be diagnosed based on the 4T's score or anti-HIT antibody if a significant reduction in platelet count combined with thrombosis is observed. Non-heparin anticoagulant drugs should be used instead of UFH in patients with strongly suspected or confirmed HIT. In contrast to UFH, LMWH can be injected subcutaneously at a dose in the target range of 0.6–1.0 IU/mL, adjusted based on the anti-Xa activity [97]. Monitoring the anti-Xa activity is crucial to prevent bleeding in patients with renal insufficiency or thrombocytopenia [86].

Fondaparinux is a synthetic anticoagulant that acts via antithrombin III to selectively inhibit the activity of factor Xa. Doses of 5, 7.5, and 10 mg for respective body weights of < 50, 50–100, and > 100 kg have been approved for the prophylaxis or treatment of VTE and acute coronary syndrome (ACS) [76]. However, fondaparinux cannot be used in patients with severe renal insufficiency (creatinine clearance rate < 30 mL/min), while the dose should be halved in patients with moderate renal insufficiency (creatinine clearance rate = 30–50 mL/min) [98]. Argatroban, a direct thrombin inhibitor, is metabolized in the liver and can significantly prolong thrombin time. The recommended infusion rate is 2 μg/ (kg·min), which can be adjusted until the APTT reaches 1.5–3 times the initial baseline value. However, an initial dose of 0.5–1.2 μg/(kg·min) is recommended for patients with moderate liver dysfunction or heart failure, while even lower initial doses 0.2–0.5 μg/(kg·min) are recommended for patients with multiple organ dysfunction [76, 82]. Bivalirudin is another direct thrombin inhibitor with short half-life (25–30 min) that can be administered to patients with HIT or ACS undergoing percutaneous coronary intervention. The recommended starting dose is 0.05 mg/ (kg·h) and should be adjusted depending on the APTT [98].

Oral anticoagulants include warfarin, rivaroxaban, apixaban, dabigatran, and edoxaban. Warfarin is a classic oral anticoagulation drug and a vitamin K antagonist that can completely inhibit the synthesis of coagulation factors II, VII, IX, and X after continuous administration for 4–5 days. In case of concomitant treatment of patients with parenteral anticoagulation and warfarin, the two therapies should overlap for at least 5 days to achieve a therapeutic INR for 24 h, at which time parenteral anticoagulant should be discontinued [72]. Dabigatran, rivaroxaban, apixaban, and edoxaban are also known as NOACs that have a short half life and can be rapidly absorbed. Dabigatran is a thrombin inhibitor, while rivaroxaban, apixaban, and edoxaban inhibit the activity of factor Xa. Patients treated with dabigatran or edoxaban should be pretreated with parenteral anticoagulant for up to 5–10 days. A higher dose is administered during the first 3 weeks of therapy for patients receiving rivaroxaban, but only during the first week for patients receiving apixaban [99].

#### Recommendation 13: the risk of bleeding needs to be assessed before initiating antiplatelet therapy in patients with trauma-induced hypercoagulopathy complicated by arterial thrombosis

Antiplatelet drugs inhibit cyclooxygenase (COX)-1, P2Y12 receptor, phosphodiesterase (PDE), or GPIIb/IIIa. This class of drugs is the first-line treatment for arterial thrombosis, but it can also increase bleeding risk in trauma patients [100]. Therefore, any antiplatelet therapy should only be planned after careful assessment of the bleeding risk in patients with trauma-induced hypercoagulopathy complicated by arterial thrombosis.

Aspirin can irreversibly inhibit the COX-1 enzyme, block the production of thromboxane, prolong the platelet production cycle, and ultimately reduce platelet aggregation. Aspirin also affects coagulation via the non-TXA2 pathway by inhibiting thrombin generation and platelet function in a dose-dependent manner and accelerating fibrinolysis [101, 102]. The plasma concentration of enteric-coated aspirin is maximal at 3–4 h after ingestion or at 30 min after chewing, and it significantly inhibits platelets within 1 h [103].

Clopidogrel and ticagrelor are the most commonly used P2Y12 receptor inhibitors. Clopidogrel, which is oxidized by the cytochrome P450 in liver enzymes, can irreversibly block the P2Y12 receptor of ADP on the platelet surface, thereby preventing activation of the ADP-mediated glycoprotein GPIIb/IIIa complex and reducing platelet aggregation. The plasma concentration of clopidogrel is maximal at 24 h with a routine dose of 75 mg, or at 4–6 h with a loading dose of 300–600 mg. Clopidogrel can also reduce platelet aggregation by 50–60%, which can return to normal within 7 days of drug withdrawal. Ticagrelor can reversibly and non-competitively bind the ADP P2Y12 receptor to reduce platelet aggregation. The platelet inhibition rate reaches 41% in 30 min and 88% in 2 h after loading of 180 mg ticagrelor, and platelet aggregation returned to normal 3–5 days after drug withdrawal. Large randomized trials support the use of ticagrelor in ACS, ischemic stroke, and peripheral vascular diseases [104]. Dual antiplatelet therapy can reduce the risk of thrombosis in patients with ACS or percutaneous coronary intervention, while aspirin is preferably combined with ticagrelor in ACS patients. In addition, clopidogrel may be used when the bleeding risk is high or the patient does not tolerate ticagrelor [105].

The most common PDE inhibitors are dipyridamole and cilostazol. Dipyridamole can inhibit the phosphodiesterase enzymes PDE-3 and PDE-5, reduce the levels of cyclic adenosine monophosphate and cyclic guanosine monophosphate, ultimately inhibiting platelet aggregation. Dipyridamole can also inhibit fibrin formation, increase extracellular adenosine levels, and cause vasodilation. The extended-release formulation of dipyridamole (200 mg) and aspirin (25 mg) is commonly used in the secondary prevention of cerebrovascular diseases [106]. Cilostazol also inhibits PDE-3 as well as platelet aggregation by reducing the expression of P-selectin. Therefore, cilostazol is mainly used to treat lower extremity arteriosclerosis and prevent stent thrombosis and stroke, while it serves as an antiplatelet therapy in case of resistance to clopidogrel [107].

The platelet–fibrinogen interaction via the GPIIb/IIIa complex is the final step in the platelet aggregation pathway. The GP IIb/IIIa inhibitors abciximab, eptifibatide and tirofiban can inhibit platelet aggregation by preventing the binding of fibrinogen to the GPIIb/IIIa complex. However, hemoglobin level and platelet count should be monitored in real time during treatment with GP IIb/IIIa inhibitors in order to closely monitor clinical bleeding.

#### Recommendation 14: interventional therapy is recommended for traumatic patients complicated by thrombosis who are at high risk of bleeding

Interventional therapy is recommended for patients with trauma-induced hypercoagulopathy complicated by thrombosis, especially when anticoagulation or thrombolysis is not effective, or the risk of bleeding is high. This type of treatment includes catheter-directed thrombolysis, percutaneous mechanical thrombectomy, venoplasty, stent implantation, and inferior vena cava filter placement. In general, interventional therapy requires lower thrombolytic drug dosages and treatment time than other therapeutic methods, while bleeding complications may also be reduced. Better thrombus clearance can also be achieved, as transcatheter administration can prevent drugs from bypassing the occluded venous segment through collateral branches, thus achieving a high drug concentration in the thrombus, while mechanical thrombectomy can improve the effectiveness of thrombolysis.

Primary percutaneous coronary intervention is recommended for patients with trauma-induced hypercoagulopathy complicated by myocardial infarction exhibiting ischemia symptoms for ≤12 h and persistent ST-segment elevation or progressive ischemia symptoms for > 12 h suggestive of hemodynamic instability or life-threatening arrhythmias [108]. Intravascular thrombectomy is recommended for patients with trauma-induced hypercoagulopathy complicated by ischemic stroke who have a middle cerebral artery occlusion within 6 h of onset and are not eligible for thrombolysis, who have a posterior circulation occlusion within 24 h of onset, or who exhibit contraindications to thrombolysis [109]. Inferior vena cava filter placement is preferred in patients with acute pulmonary embolism and anticoagulant contraindications to prevent DVT in lower extremities [76]. Catheter-directed thrombolysis is recommended for patients with intracranial venous thrombosis who have no intracranial hemorrhage and who do not respond to anticoagulant therapy, as well as for DVT patients whose acute iliofemoral venous thrombosis symptoms last fewer than 14 days and who are at high risk of recurrence [110, 111]. Mechanical thrombectomy is recommended for patients with intracranial venous thrombosis complicated by progressive symptoms after anticoagulation therapy, new bleeding after thrombolytic therapy, or severe intracranial hemorrhage at admission. Both catheter-directed thrombolysis and mechanical thrombectomy are also recommended for patients with severe acute pulmonary embolism who have thrombolytic contraindication or are at high risk of bleeding [110].

#### Recommendation 15: routine systemic thrombolysis is not recommended for patients with trauma-induced hypercoagulopathy complicated by thrombosis

Systemic thrombolysis refers to intravenous injection of thrombolytic drugs away from the target vessel and its absolute contraindications include active hemorrhage, recent skull fracture, recent brain or spinal cord surgery, ischemic stroke within 3 months of onset, intracranial structural lesions, and aortic dissection [112]. Although successful systemic thrombolysis can rapidly dissolve the thrombus and restore blood supply to tissues, it can also increase the bleeding risk. Therefore, trauma-induced hypercoagulopathy is considered another contraindication to systemic thrombolytic therapy, which can be performed without interventional therapy only in patients with trauma-induced hypercoagulopathy complicated by acute life-threatening thrombosis [96]. However, cautious risk-benefit analysis is required prior to treatment, including the risk of thrombosis-related mortality and the risk of bleeding associated with thrombolytic therapy.

## Conclusions

Trauma-induced hypercoagulopathy is a TIC hypercoagulable phenotype induced by tissue injury, inflammation and stress, which may transform into hypocoagulable phenotype with increasing blood loss. If trauma patients are diagnosed as hypercoagulable state by viscoelastic coagulation tests, mechanical prevention alone or combined with pharmacologic prophylaxis should be planned for preventing thrombosis. If patients with trauma-induced hypercoagulopathy are complicated by thrombosis, the risk of bleeding needs to be assessed before initiating individualized antithrombotic or interventional therapy.

## Data Availability

Not applicable.

## Abbreviations

ACS: Acute coronary syndrome
ADP: Adenosine diphosphate
APC: Activated protein C
APLS: Antiphospholipid antibody syndrome
APTT: Activated partial prothrombin time
AT: Antithrombin
CALR: Calreticulin
CBC: Complete blood count
COX: Cyclooxygenase
DD: D-dimer
DIC: Disseminated intravascular coagulation
DVT: Deep venous thrombosis
FDPs: Fibrinogen degradation products
HIT: Heparin-induced thrombocytopenia
INR: International standardized ratio
ISS: Injury severity score
JAK: Janus kinase
LA: Lupus anticoagulant
LDH: Lactate dehydrogenase
LMWH: Low molecular weight heparin
MA: Maximum amplitude
NOAC: New oral anticoagulants
PAI-1: Plasminogen activator inhibitor type-1
PDE: Phosphodiesterase
PNH: Paroxysmal nocturnal hemoglobinuria
SLE: Systemic lupus erythematosus
TEG: Thrombelastography
TIC: Trauma-induced coagulopathy
t-PA: Tissue-type plasminogen activator
PT: Prothrombin time
TT: Thrombin time
TXA: Tranexamic acid
UFH: Unfractionated heparin
VKA: Vitamin K antagonist
VTE: Venous thromboembolism
